# Can North American animal poison control center call data provide early warning of outbreaks associated with contaminated pet food? Using the 2007 melamine pet food contamination incident as a case study

**DOI:** 10.1371/journal.pone.0277100

**Published:** 2022-12-08

**Authors:** Alexandra L. Swirski, David L. Pearl, Olaf Berke, Terri L. O’Sullivan

**Affiliations:** Department of Population Medicine, University of Guelph, Guelph, Ontario, Canada; Zagazig University, EGYPT

## Abstract

The 2007 melamine pet food contamination incident highlighted the need for enhanced reporting of toxicological exposures and development of a national quantitative disease surveillance system for companion animals. Data from poison control centers, such as the Animal Poison Control Center (APCC), may be useful for conducting real-time surveillance in this population. In this study, we explored the suitability of APCC call data for early warning of toxicological incidents in companion animal populations by using a-priori knowledge of the melamine-related nephrotoxicosis outbreak. Patient and household-level information regarding possible toxicological exposures in dogs and cats reported to the APCC from 2005 to 2007, inclusive, were extracted from the APCC’s AnTox database. These data were used to examine the impact of surveillance outcome, statistical methodology, analysis level, and call source on the ability to detect the outbreak prior to the voluntary recall issued by the pet food manufacturer. Retrospective Poisson temporal scan tests were applied for each combination of outcome, method, level, and call source. The results showed that month-adjusted scans using syndromic data may have been able to help detect the outbreak up to two months prior to the voluntary recall although the success of these methods varied across call sources. We also demonstrated covariate month-adjustment can lead to vastly different results based on the surveillance outcome and call source to which it is applied. This illustrates care should be taken prior to arbitrarily selecting a surveillance outcome and statistical model for surveillance efforts and warns against ignoring the impacts of call source or key covariates when applying quantitative surveillance methods to APCC call data since these factors can lead to very different results. This study provides further evidence that APCC call data may be useful for conducting surveillance in the US companion animal population and further exploratory analyses and validation studies are warranted.

## Introduction

There have been vast improvements in animal disease surveillance methodology over the past decades due, in part, to the adoption of electronic medical records and online reporting, coupled with substantial advancements in computing power and the development of geographic information systems. Consequently, there has been an increase in the implementation of surveillance systems in both human and animal populations [1–5]. Historically, surveillance systems in animal populations have primarily focused on livestock species [4, 5]. However, the 2007 melamine pet food contamination incident, which impacted companion animal populations in North America, Europe, and South Africa, highlighted the need for enhanced reporting of toxicological exposures in companion animals and the development of a real-time syndromic surveillance system to help prompt veterinary and human public health action during future outbreaks [6–9].

Syndromic surveillance uses existing data from non-traditional sources to categorize groups of similar signs/symptoms or related metrics into ‘syndromes’, to identify disease clusters before diagnoses are confirmed and reported, thereby identifying outbreaks earlier than non-syndromic methods [10]. Although it began as a method for the early detection of bioterrorism incidents in human populations [10], syndromic surveillance has been successfully applied in a veterinary medicine context for the surveillance of livestock populations over the past 20 years [4, 11–13]. Recently, syndromic surveillance has been used in companion-animal populations to successfully identify both infectious and non-infectious disease outbreaks, such as accidental environmental releases of toxic chemicals [14], clusters of infectious disease [15], and foodborne outbreaks [16, 17].

A variety of statistical methods exist for the identification of unusual events or patterns in syndromic surveillance data [10, 18–20]. Recently, cluster detection methods (e.g., scan statistics) have been applied to syndromic surveillance data as an approach to identify potential outbreaks of disease in which cases cluster in space and/or time [12, 15, 21]. Scan statistics are particularly suited to this application since they can be used to identify spatial, temporal, and spatial-temporal clusters of a syndrome, which may signal a potential outbreak incidents, and analyses may be conducted both retrospectively and prospectively [21, 22]. Scan statistics have been effectively used both with [12, 21] and without covariate adjustment [23, 24]. However, recent work has shown that covariate-adjustment can improve the performance of scan tests as a surveillance tool by removing the “noise” of predictable covariates, thereby reducing the number of false alarms [12, 21].

Currently, there is no centralized system for reporting diseases and toxicological exposures in companion animals in North America [25]. Consequently, syndromic surveillance initiatives have primarily utilized electronic medical record data from veterinary hospitals and clinics [4, 5, 15, 16]. However, poison centers (PCs) represent a novel and under-utilized data source for conducting real-time syndromic surveillance of toxicological exposures in pets [14].

Several animal-specific PCs currently exist within the US for seeking assistance with toxicological exposures involving pets. One such PC, the Animal Poison Control Center (APCC), is a 24-hour animal PC operated by the American Society for the Prevention of Cruelty to Animals (ASPCA). The APCC operates under a cost-recovery model and charges a small one-time consult fee for each suspected poisoning incident at the household level. The APCC receives a large volume of calls from across the United States (US) concerning toxicological exposures in companion animals and collects extensive data from each call. Call data are subsequently stored in the Animal Toxin (AnTox) database. Call data extracted from AnTox have been successfully used by the APCC to identify emerging problems in toxicology [26, 27]. These characteristics make the APCC an ideal candidate for exploring the suitability of PC data for the development of a real-time quantitative syndromic surveillance system for monitoring toxicological exposures in the US companion animal population.

Therefore, the goal of this study was two-fold. First, to use APCC call data from 2005 to 2007 and *a priori* knowledge of the 2007 melamine pet food contamination incident as a case study to explore the suitability of PCC data for conducting surveillance in companion animal populations to enable early detection of large-scale pet food contamination incidents using known statistical methods (i.e., temporal scan statistics) that have been successfully applied to syndromic surveillance efforts in other populations [1]. Second, to compare the performance of different parameterizations of the temporal scan statistics to identify this contamination incident retrospectively. The specific objectives of this goal were as follows:

- Assess if applying temporal scan statistics to APCC call data could have successfully identified the 2007 melamine contamination incident prior to the voluntary recall by the pet food manufacturer;
- Compare the performance of temporal scan statistics with and without adjustment for background levels of calls to retrospectively detect the 2007 melamine contamination incident prior to the voluntary recall by the pet food manufacturer; and
- Explore the impact of surveillance outcome (i.e., syndrome vs. exposure), source of call (i.e., general public vs. veterinarians), and level of analysis (i.e., patient vs. household) on the performance of the retrospective scans.

## Materials and methods

### Case study: 2007 melamine contamination incident

**Fig 1:**
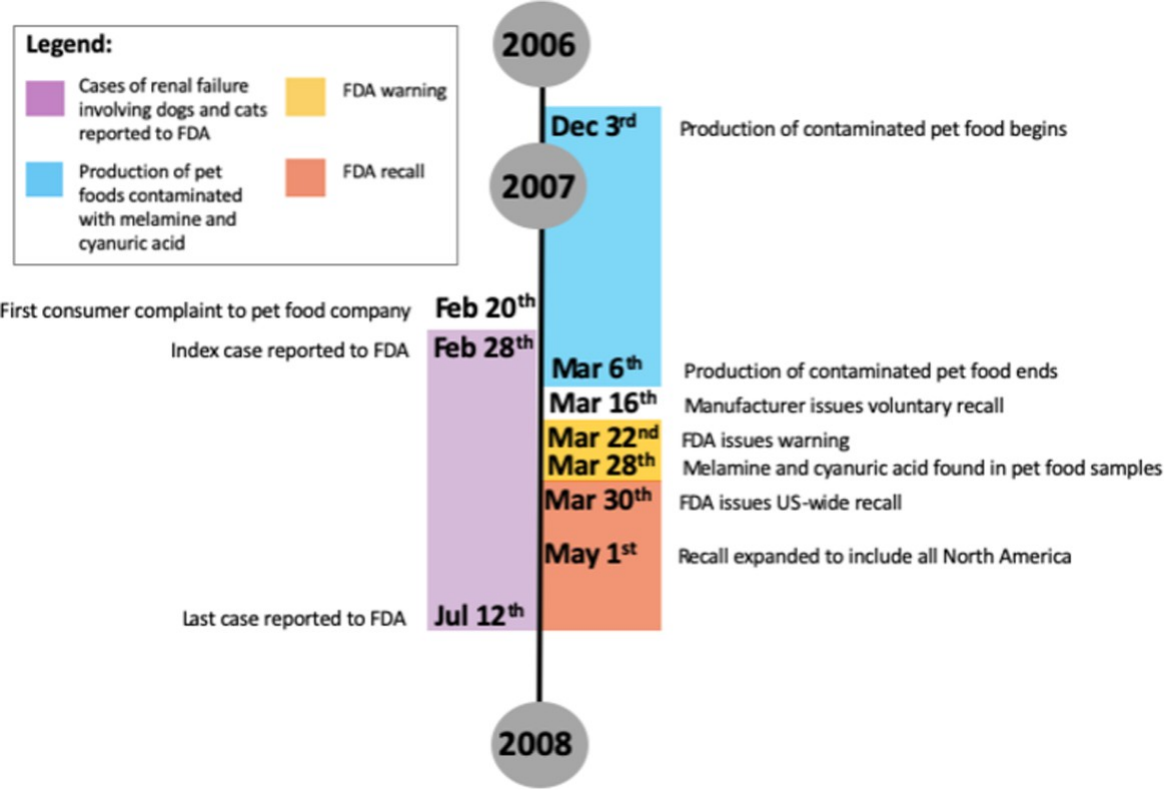
A timeline of the 2007 melamine pet food contamination outbreak in North America.

In early 2007, a point-source foodborne illness outbreak of acute nephrotoxic renal failure was identified in dogs and cats in North America [8, 9, 28]. This outbreak was a consequence of dog and cat exposures to pet foods manufactured with wheat gluten adulterated with melamine and cyanuric acid, a melamine analogue [8, 9, 28]. The contaminated pet food products were produced between December 3, 2006, and March 6, 2007. Cases of renal failure were reported to the FDA, with the index case reported on February 28, 2007 and the final case on July 12, 2007 (Fig 1) [7, 8]. Estimates of the number of pets impacted by this outbreak vary substantially, but one survey of laboratories and veterinarians reported 424 confirmed cases (66% cats and 34% dogs) [29]. A-priori knowledge of this outbreak was used to explore if call data regarding possible toxicological exposures involving dogs and cats from the APCC could have been used to identify this outbreak prior to the voluntary food recall by the manufacturer on March 16, 2007.

### Data sources and variables

Patient and household-level information regarding possible toxicological exposures in dogs and cats reported to the APCC from the contiguous US from 2005 to 2007, inclusive, were extracted from the AnTox database. Data extracted included: source of call (i.e., public, veterinarian, other poison control center), call information (i.e., location, time, payment), patient characteristics (e.g., species), exposure (i.e., pet foods and treats, other exposures) and clinical findings. Data concerning clinical findings were extracted using two variables: `measure type’ and `finding area’. `Measure type’ refers to the type of measure reported for clinical signs and diagnostic test results (e.g., vomiting, lethargy, elevated kidney values, serum liver enzymes, ataxia). However, this variable does not capture the value or units for each finding. `Finding area’ corresponds to the area of the body or body system each measure type involves and is categorized by APCC staff. For example, anorexia would be classified as ‘General Disorders’, serum iron would be classified as ‘Serum’, and polyuria would be classified as ‘Urinary’.

Calls originating from the American Product Safety Service, were excluded from all analyses since they are not representative of normal use of the hotline; the American Product Safety Service is administered by the APCC but funded by companies to support calls related specifically to their products.

A ‘call’ was defined as a single suspected exposure incident in an individual household that resulted in a call to the APCC and may have involved more than one animal or substance. As owners and veterinarians are encouraged to call back to report findings or outcomes, only the first call to the APCC was counted per exposure incident, in order to avoid double counting. However, if the same household called and reported a different incident, it would have been classified as a separate call and subsequently counted. For all subsequent discussions, a ‘household-level’-analysis refers to analysis at the call-level and may have involved more than one animal or substance. A ‘patient’ was defined as a single animal involved in a suspected exposure incident in an individual household and may have been exposed to more than one substance.

### Surveillance outcomes

To identify the most effective method for early detection of the melamine outbreak, two different disease surveillance outcomes were compared: exposure-based surveillance and syndromic surveillance. Exposure-based surveillance used exposures to dog and cat food products as the outcome. In contrast, syndromic surveillance used two syndromes, renal dysfunction syndrome (RDS) and melamine-cyanuric acid toxicosis syndrome (MCTS), as outcomes for analysis. After classification of each exposure/syndrome was completed, data subsets were created for each measure for subsequent analyses.

### Exposures to dog and cat foods

Exposures to dog and cat food products were identified based on the product name and primary ingredient for each suspected exposure reported for each patient. As some patients may have been exposed or suspected to have been exposed to many substances, an animal was considered exposed to pet food products if they had been reported to be exposed to at least one pet food product. Exposures to pet food products intended for consumption by other species (e.g., pocket pets, birds, fish, amphibians) were excluded.

#### Syndromic surveillance

Within 12 hours of exposure to toxic amounts of melamine and cyanuric acid, dogs and cats generally develop inappetence with or without vomiting [9, 30, 31]. Once renal dysfunction develops, the animal may show other non-specific signs of illness as a consequence of renal dysfunction, such as weakness, lethargy, and dehydration [9, 28]. In severe cases, exposure to toxic levels of melamine and cyanuric acid can cause acute renal failure in dogs and cats and laboratory findings from animals presenting with melamine-cyanuric acid toxicosis are generally consistent with acute renal failure [9, 28, 30, 31].

We explored two different syndrome definitions, based on measures categorized by APCC staff during consult calls and captured in the ‘measure type’ and ‘finding area’ variables within the AnTOX database. The measure type contains information on the clinical sign found and may include aggregate outcomes, as classified by APCC staff. For example, a measure type of ‘renal azotemia’ could correspond to elevated kidney values, specifically blood urea nitrogen and creatine. The ‘finding area’ refers to the body system that clinical sign is linked to. These two variables were chosen to explore the feasibility of conducting surveillance using pre-defined measures within the AnTOX database. The two syndromic definitions used for analysis were as follows:

- Renal dysfunction syndrome (RDS)
  The RDS definition was based on the urinary category of the ‘finding area’ variable. This syndrome included all measure types which were assigned by APCC staff to the urinary finding area. Examples of measure types captured by this syndrome include renal failure, polydipsia, hyperkalemia, and elevated kidney values. All patients where at least one urinary finding area was reported were classified as an RDS case.
- Melamine-cyanuric acid toxicosis syndrome (MCTS)
  The MCTS definition was developed based on reported clinical signs and findings of toxicosis associated with exposure to toxic amounts of melamine and cyanuric acid in dogs and cats, as listed in Table 1 and used the `measure type’ variable within AnTOX [9, 28, 30, 31]. A patient was classified as a MCTS case if they had at least one measure reported from the measure types included in the MCTS syndrome definition.

**Table 1:** Measure types included in the melamine-cyanuric acid toxicosis syndrome (MCTS) definition for dogs and cats.

Measure types included for both cats and dogs	Measure types included for cats	Measure types included for cats
• Diarrhea	• Measurement of urine specific gravity	• Renal azotemia
• Anorexia		• Leukocytes
• Vomiting		• Neutrophilia
• Lethargy		
• Renal failure		
• Acute renal failure		
• Weakness		
• Urine potassium		
• Hyperkalemia		
• Hyperphosphatemia		
• Elevated kidney values		
• Not urinating		
• Polydipsia		
• Polyuria		
• Elevated creatine		

Note: MCTS syndromic components were chosen based on documented clinical signs and laboratory findings associated with dog and cat exposures to toxic amounts of melamine and cyanuric acid [9, 28, 30, 31]. Although some of these measure types may represent aggregate outcomes, for example, renal azotemia corresponds to elevated kidney values specifically blood urea nitrogen and creatinine, these measures were categorized by APCC staff during the call and these pre-classified measures were used for analysis.

As some measure types overlapped between the RDS and MCTS definitions, a patient could be included in analyses for both syndromes. For example, an animal with acute renal failure would be included in analyses for both syndromes, but a lethargic animal would have only been included in the MCTS analysis.

### Retrospective scan statistics

Retrospective temporal scan statistics were used to explore if it was possible to retrospectively identify the 2007 melamine contamination incident prior to the voluntary pet food recall by the pet food manufacturer on March 16, 2007, using APCC call data. Two different methodological approaches were compared to identify which method would perform better at identifying clusters of syndromes: an unadjusted Poisson purely temporal scan statistic, and a model-adjusted Poisson purely temporal scan statistic.

SatScan™ software (v.9.6) was used for all retrospective temporal scans, using data from 2007 [32]. The following parameters were consistent for all temporal scans: the maximum temporal scanning window was set to the maximum allowable level (i.e., 50% of the study period); one-sided scans for high rates were conducted. A Monte Carlo simulation-based p-value was used for comparison with a significance level of 5% to determine the statistical significance of each cluster. However, it is important to note that this term is used in an exploratory rather than confirmatory sense throughout this manuscript due to concerns surrounding the misuse of the term “statistically significant” [33]. Monte Carlo simulations were performed with 9999 replications and a relative rate was reported for each cluster to measure how many times greater the rate within the scanning window was than the rate outside of the window [22, 34].

Unadjusted Poisson temporal scans used raw counts of the number of households or patients reported to the APCC that matched the outcome measure being examined (i.e., exposure to pet foods, RDS, and MCTS cases). An offset was not employed for these scans.

#### Month-adjusted temporal scans

The model-adjusted Poisson temporal scans used the same outcomes as the unadjusted scans. However, previous work has identified that rates of calls to the APCC are heavily influenced by season and vary by species [35]. To control for the effect of month, univariable Poisson regression models, one for each outcome measure were fitted using STATA® v.15 for Windows (Stata Corporation, College Station, Texas, US) to examine the association between month and the number of calls to the APCC, using data from the two years prior to the melamine contamination incident (i.e., 2005 and 2006). The scan test was based on the counts of calls for each day, after controlling for the number of expected daily counts as the offset, as predicted by the Poisson regression model.

#### Impact of data aggregation and caller source

Subsets of the datasets created for each surveillance outcome were used to further examine the impact of the level of data aggregation and caller source, as follows:

- Call source
  First, calls were classified by their source into three categories: calls from all sources (e.g., public, veterinarians, other poison control centers, other), calls from the general public, and calls from veterinarians. This classification was based on the individual who made first contact with the APCC and provided basic information about the animal and the suspected exposure. If multiple sources were recorded from a household, the source for the first case from the household was recorded.
- Level of data aggregation
  Two subsets of data were created using the call source classification for each surveillance outcome. The first subset was created by aggregating the calls at the household-level (i.e., multiple animals could have been involved in an exposure that occurred in one household). The second subset was created by aggregating calls at the patient-level.

**Fig 2:**
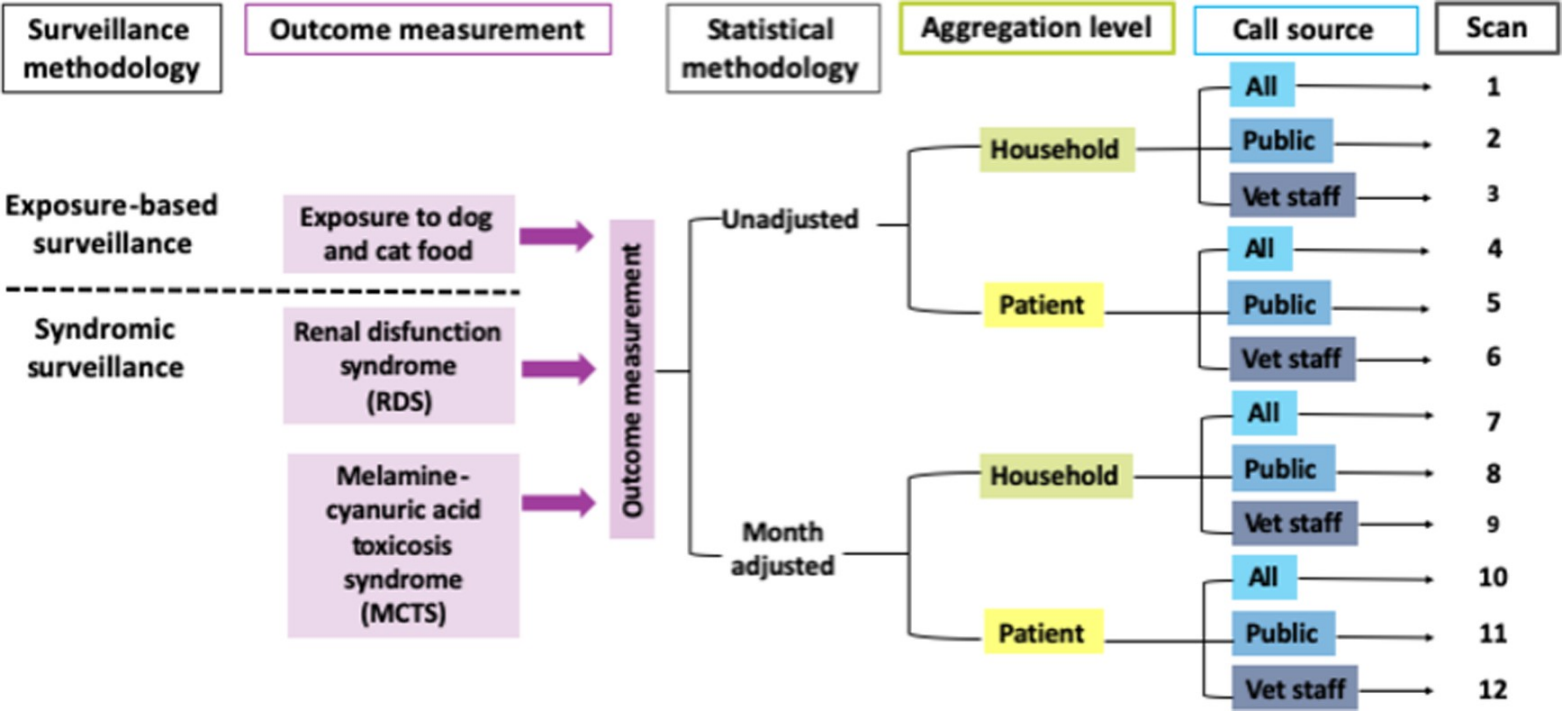
Flow chart of the 12 scans conducted for each outcome measure and combination of surveillance outcome, statistical methodology, data aggregation level and call source.

Retrospective temporal scans were subsequently run for each combination of surveillance outcome, methodology, call source, and aggregation level, resulting in 12 scans for each outcome, as illustrated in Fig 2. The most likely statistically significant cluster with high rates of calls for each scan were reported.

## Results

From 2005 to 2007, inclusive, the APCC received 61,741 calls regarding suspected exposures to potentially toxic substances involving 54,640 dogs and 9,739 cats. Of these calls, 63.4% (n = 39,123) originated from the general public, 36.6% (n = 22,611) came from veterinarians, and the remaining seven calls came from other or unknown sources.

### Exposure-based surveillance

Dog and cat exposures to pet food products were reported in 241 (0.4%) of the 26,205 calls received by the APCC from 2005 to 2007 and involved 236 dogs and 27 cats (Table 2). Calls regarding pet food exposures predominantly originated from the general public for calls involving dogs (Table 2). Whereas, there were approximately an even number of calls from the public or veterinarians for pet food exposures involving cats (Table 2).

**Table 2:** Summary of the number and percent of dogs and cats involved in suspected exposures to potentially toxic substances reported to the APCC^1^ from 2005 to 2007, by surveillance outcome and call source.

Outcome	Call source^2^	Dogs		Cats	
		No. of patients [Percentage of total]	No. of calls [Percentage of total]^8^	No. of patients [Percentage of total]	No. of calls [Percentage of total]^8^
**Exposure to pet food products**	Public	179 [0.5%]	160 [0.5%]	14 [0.3%]	14 [0.3%]
	Veterinary staff	57 [0.3%]	53 [0.3%]	13 [0.3%]	13 [0.3%]
	All sources^3^	236 [0.4%]	213 [0.4%]	27 [0.3%]	27 [0.3%]
**Renal dysfunction syndrome** ^4^	Public	2,264 [6.4%]	2,168 [6.4%]	274 [4.8%]	270 [5.0%]
	Veterinary staff	910 [4.7%]	893 [4.8%]	196 [4.8%]	195 [5.0%]
	All sources	3,175 [5.8%]	3,062 [5.8%]	470 [4.8%]	465 [5.0%]
**Melamine-cyanuric acid toxicosis syndrome** ^5^	Public	11,790 [33.5%]	11,398 [33.8%]	1,749 [30.9%]	1,682 [31.0%]
	Veterinary staff	5,640 [29.1%]	5,512 [29.5%]	1,222 [30.0%]	1,195 [30.6%]
	All sources	17,430 [31.9%]	16,910 [32.9%]	2,971 [30.5%]	2,877 [30.8%]
**Total** ^**6**^ ^**,**^ ^**7**^	Public	35,234 [64.5%]	33,719 [64.3%]	5,666 [58.2%]	5,425 [58.1%]
	Veterinary staff	19,400 [35.5%]	18,705 [35.7%]	4,073 [41.8%]	3,909 [41.9%]
	All sources	54,640	52,430	9,739	9,335

^1^The APCC [Animal Poison Control Center) is a 24-hour animal poison control center operated by the American Society for the Prevention of Cruelty to Animals. Analyses only included data originating from the contiguous US.

^2^Call source was based on the individual who made the first contact with the APCC for a patient and provided patient demographic information [breed, sex, age). For calls involving multiple species, call source was based on the first call for that household.

^3^All sources category includes calls from the public, veterinarians, other poison control centers, other sources, and unknown sources.

^4^**Renal dysfunction syndrome:** syndrome grouping based on the urinary category used by the APCC staff and includes measures such as renal failure, polydipsia, hyperkalemia, and elevated kidney values. All patients with at least one urinary finding area were classified as a renal dysfunction syndrome case.

^5^**Melamine-cyanuric acid toxicosis syndrome:** syndrome grouping based on reported clinical signs and laboratory findings associated with dog and cat exposures to toxic amounts of melamine and cyanuric acid [9, 28, 30, 31].

^6^**Total patients:** refers to the number of patients involved in suspected exposures to potentially toxic substances reported to the APCC during toxicological consult calls.

^7^**Total calls:** refers to the number of calls to the APCC regarding suspected exposures to potentially toxic substances.

^8^**Percent of total patients:** this is the percentage out of all the patients/calls originating from that call source.

**Fig 3:**
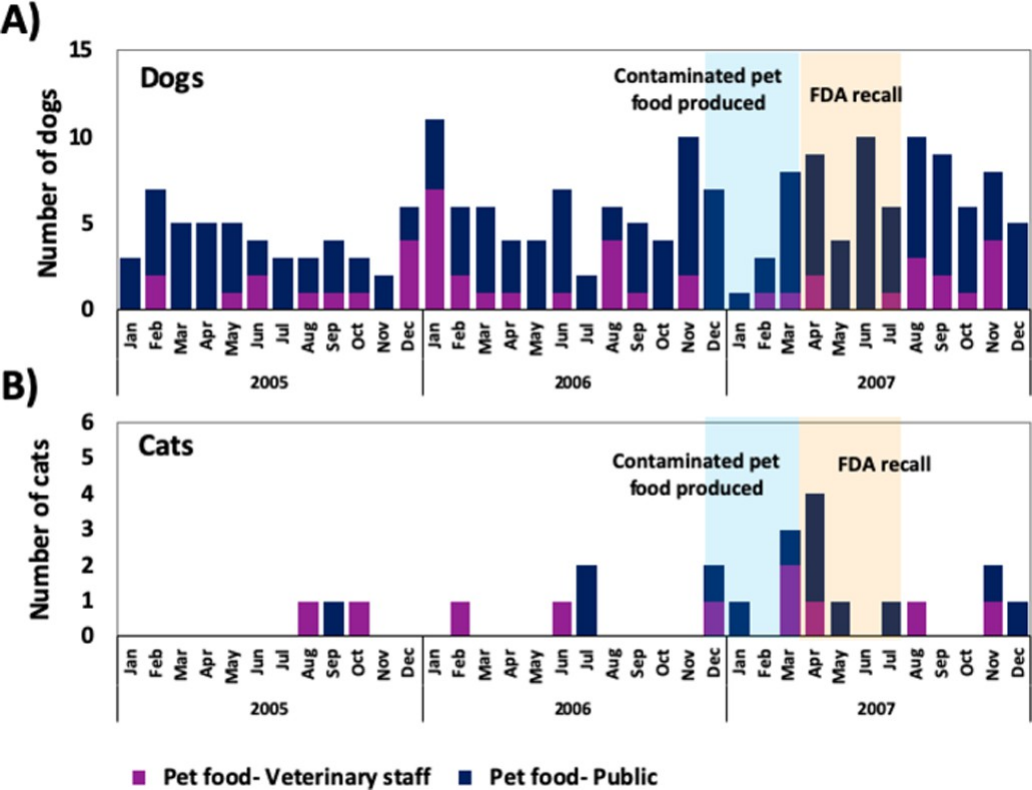
Number of dogs and cats exposed to pet food products, by call source, as reported to the APCC during toxicological consult calls from 2005 to 2007, inclusive. (A) Number of dogs. (B) Number of cats.

Out of the three study years, 2007 had the highest number of calls involving pet food exposures (n = 108; 0.4% of total calls), compared to the two previous years (2005 (n = 64; 0.4%), 2006 (n = 91; 0.4%)), however, the proportion of calls involving pet food was approximately even among the three years (Fig 3).

#### Unadjusted retrospective temporal scans

Scans of the entire dataset or the subset of calls from the public involving dogs and cats identified statistically significant high-rate clusters of calls to the APCC involving pet food products with start dates prior to the voluntary recall on March 16, 2007 (Table 3). Scans of the entire dataset identified a significant high-rate household-level cluster from March 13 to April 23 and a high-rate patient-level cluster from March 13 to September 10 (Table 3). Scans of calls from the public identified a significant high-rate household-level cluster from March 13 to June 18 and a patient-level cluster from March 13 to September 10 (Table 3). No statistically significant clusters were identified for scans of calls from veterinarians (Table 3).

**Table 3:** Statistically significant results of unadjusted retrospective temporal scan statistics using a Poisson model examining calls to the APCC^1^ involving reported dog and cat exposures to pet food products (January 1 –December 31, 2007).

Call source	Analysis level	Date of most likely cluster	Number of cases	Number of expected cases	Relative Risk	*P*-value^✢^
**All**	Household	Mar 13 –Apr 23*	26	11.6	2.7	0.009
	Patient	Mar 13 –Sep 10*	74	53.9	2.2	0.009
**Public**	Household	Mar 13– Jun 18*	37	20.1	2.7	0.008
	Patient	Mar 13 –Sep 10*	59	40.9	2.6	0.006
**Veterinary staff**	Household	No significant cluster				
	Patient	No significant cluster				

* Indicates cluster detected with a start date prior to voluntary recall on March 16, 2007.

^**✢**^ Monte Carlo (9999 replications) based *P*-value.

^1^The APCC (Animal Poison Control Center) is a 24-hour animal poison control center operated by the American Society for the Prevention of Cruelty to Animals. Analyses only included data originating from the contiguous US.

#### Month-adjusted retrospective temporal scans

Results of the month-adjusted retrospective scans identified statistically significant high-rate clusters prior to March 16, 2007 for scans of the entire dataset and calls from the public regardless of analysis level (Table 4). Scans of the entire dataset identified a significant high-rate household-level cluster from March 13 to April 23 and a high-rate patient-level cluster from March 13 to September 10 (Table 4). Similarly, scans of calls from the public identified a significant high-rate household-level cluster from March 13 to June 18 and a patient-level cluster from March 13 to September 10 (Table 4). Scans of calls from veterinary staff identified two statistically significant high rate clusters, but neither were found prior to March 16 (Table 4).

**Table 4:** Statistically significant results of month-adjusted^1^ retrospective temporal scan statistics using a Poisson model examining calls to the APCC^2^ involving reported dog and cat exposures to pet food products (January 1 –December 31, 2007).

Call source	Analysis level	Date of most likely cluster	Number of cases	Number of expected cases	Relative Risk	*P*-value^✢^
**All**	Household	Mar 13 –Apr 23*	24	9.8	3.2	0.002
	Patient	Mar 13 –Sep 10*	74	49.3	2.6	0.001
**Public**	Household	Mar 13 –Jun 18*	37	20.0	2.7	0.006
	Patient	Mar 13 –Sep 10*	59	39.1	2.8	0.002
**Veterinary staff**	Household	Mar 20 –Apr 9	6	0.4	17.6	0.001
	Patient	Mar 20 –Apr 9	6	0.4	19.4	0.001

* Indicates cluster detected with a start date prior to voluntary recall on March 16, 2007.

^**✢**^ Monte Carlo (9999 replications) based *P*-value.

^1^In order to control for the effect of month, univariable Poisson models were fitted for each outcome using data from the two years prior to the melamine contamination incident (i.e., 2005–2006). The number of expected calls per day were predicted from each model after controlling for month and subsequently used as the denominator for the month-adjusted scans.

^2^The APCC (Animal Poison Control Center) is a 24-hour animal poison control center operated by the American Society for the Prevention of Cruelty to Animals. Analyses only included data originating from the contiguous US.

### Syndromic surveillance

**Fig 4:**
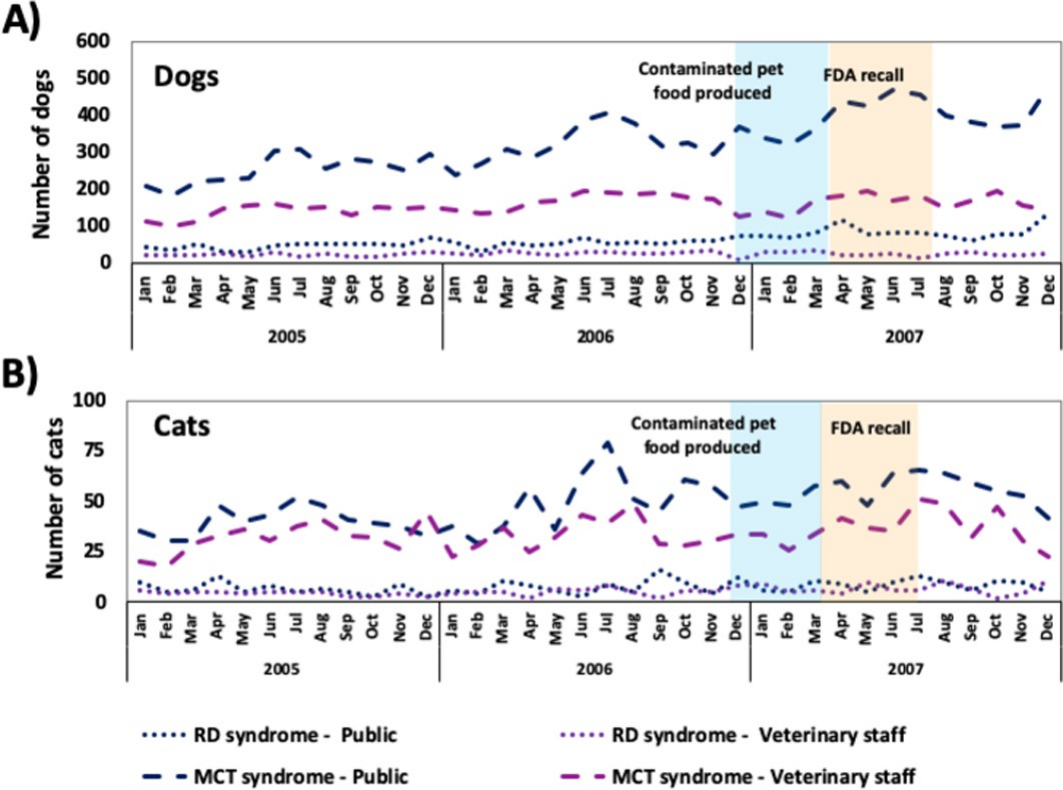
Number of dogs and cats with urinary and renal failure syndromes reported to the APCC during a toxicological consult call from 2005 to 2007, inclusive, stratified by call source. (A) Number of dogs. (B) Number of cats.

Throughout the entire study period, 5.8% and 31.9% of dogs and 4.8% and 30.5% of cats were classified into the RDS and MCTS groupings, respectively (Table 2). For both syndrome definitions, approximately 40% of calls to the APCC involving pets classified into either syndrome group occurred in 2007, for both species (Fig 4).

#### Unadjusted retrospective temporal scans

Within the RDS data, unadjusted retrospective temporal scans identified four statistically significant high-rate clusters, but there were no clusters found prior to March 16 (Table 5).

**Table 5:** Results of unadjusted retrospective temporal scan statistics using a Poisson model examining renal dysfunction syndrome (RDS) and melamine-cyanuric acid toxicity syndrome (MCTS) calls involving dogs and cats to the APCC^1^ (January 1 –December 31, 2007).

Syndrome definition	Analysis level	Call source	Date of most likely cluster	Number of cases	Number of expected cases	Relative Risk	*P*-value^✢^
**RDS**	Household	All	Dec 18–31	95	55.8	1.8	0.002
		Public	Dec 18–31	78	41.3	2.0	0.001
		Veterinary staff	No significant clusters				
	Patient	All	Dec 18–31	102	58.0	1.8	0.001
		Public	Dec 18–31	84	43.1	2.0	0.001
		Veterinary staff	No significant clusters				
**MCTS**	Household	All	Mar 20 –Aug 13	3371	3076.9	1.2	0.001
		Public	Mar 20 –Sep 3	2653	2431.2	1.2	0.001
		Veterinary staff	Mar 13 –Aug 13*	1084	996.2	1.2	0.04
	Patient	All	Mar 20 –Aug 13	3496	3185.3	1.2	0.001
		Public	Mar 20 –Sep 3	2755	2526.9	1.2	0.001
		Veterinary staff	Mar 13 –Aug 13*	1117	1020.6	1.2	0.01

* Indicates cluster detected with a start date prior to voluntary recall on March 16, 2007.

^**✢**^ Monte Carlo (9999 replications) based *P*-value.

^1^The APCC (Animal Poison Control Center) is a 24-hour animal poison control center operated by the American Society for the Prevention of Cruelty to Animals. Analyses only included data originating from the contiguous US.

Within the MCTS data, six statistically significant high-rate clusters were identified, but only scans of calls from veterinary staff identified significant clusters prior to March 16 (Table 5). Scans of calls from veterinarians identified a significant high-rate cluster from March 13 to August 13 for both household and patient-level scans (Table 5).

#### Month-adjusted retrospective temporal scans

Within the RDS data, month-adjusted scans identified four statistically significant high-rate clusters (Table 6). Two significant high-rate clusters were identified from January 30 to May 28 by scanning calls from all sources at both the household and patient levels (Table 6). These clusters were detected prior to the first consumer complaint to the pet food company on February 20.

**Table 6:** Results of month-adjusted^1^ retrospective temporal scan statistics using a Poisson model examining renal dysfunction syndrome (RDS) and melamine-cyanuric acid toxicosis syndrome (MCTS) calls involving dogs and cats to the APCC^2^ (January 1 –December 31, 2007).

Syndrome definition	Analysis level	Call source	Date of most likely cluster	Number of cases	Number of expected cases	Relative Risk	*P*-value^✢^
**RDS**	Household	All	Jan 30 –May 28**	498	416.5	1.3	0.002
		Public	Mar 20 –Apr 30	166	111.5	1.6	0.001
		Veterinary staff	No significant clusters				
	Patient	All	Jan 30 –May 28**	518	434.8	1.3	0.002
		Public	Mar 27 –Apr 30	148	94.6	1.7	0.001
		Veterinary staff	No significant clusters				
**MCTS**	Household	All	Jan 30 –May 28**	2993	2725.6	1.2	0.001
		Public	Jan 1 –May 28**	2048	1849.39	1.2	0.001
		Veterinary staff	No significant clusters				
	Patient	All	Jan 1 –May 28**	3090	2814.5	1.2	0.001
		Public	Jan 1 –May 28**	2126	1923.5	1.2	0.001
		Veterinary staff	May 1 –Oct 29	1291	1153.9	1.3	0.001

* Indicates cluster detected with a start date prior to voluntary recall on March 16, 2007.

** Indicates cluster detected with a start date prior to first consumer complaint to pet food company on February 20, 2007.

^**✢**^ Monte Carlo (9999 replications) based *P*-value.

^1^In order to control for the effect of month, univariable Poisson models were fitted for each outcome using data from the two years prior to the melamine contamination incident (i.e., 2005–2006). The number of expected calls per day were predicted from each model after controlling for month and subsequently used as the denominator for the month-adjusted scans.

^2^The APCC (Animal Poison Control Center) is a 24-hour animal poison control center operated by the American Society for the Prevention of Cruelty to Animals. Analyses only included data originating from the contiguous US.

Within the MCTS syndrome data, five statistically significant high-rate clusters were identified, four of which were identified prior to the voluntary recall on March 16 (Table 6). Two significant high-rate clusters were identified from January 1 to May 28 by scanning calls from the public at both the household and patient-level (Table 6). Scans of the entire dataset at patient-level also identified a significant high-rate cluster from January 1 to May 28 (Table 6). A statistically significant high-rate cluster was identified from January 30 to May 28 by scanning the entire dataset at the household-level (Table 6).

## Discussion

The 2007 global outbreak of melamine contaminated pet food emphasized the need for enhanced reporting of toxicological exposures in companion animals and development of a syndromic disease surveillance system to initiate veterinary and human public health action during future large-scale toxicant exposure incidents. However, conducting surveillance in this population is particularly challenging as a centralized system for reporting diseases and toxicological exposures in companion animals currently does not exist at the national-level in North America [25]. In addition, companion animal population estimates, or censuses are not readily available for use which makes identifying an appropriate denominator difficult. Although surveillance initiatives in this population have primarily utilized EMR data from veterinary hospitals and clinics [4, 5, 15, 16], this study demonstrates that call data from the APCC may be a useful alternative data source for quantitative disease surveillance of toxicological incidents in the US companion-animal population. However, there are inherent characteristics in PC data, especially in a companion-animal context, that may bias the results of quantitative cluster detection methods and should be examined prior to use.

Several aberration and cluster detection methods exist for the identification of unusual events or patterns in surveillance data that may be suitable for use with APCC call data. In this study we compared the performance of temporal scan statistics [32] using different outcome measures, adjustment for expected call number, and call source, to retrospectively detect the 2007 melamine contamination incident prior to the voluntary recall by the pet food manufacturer. Scan statistics were chosen since they are particularly useful for quantitative disease surveillance; they provide a flexible scanning window, useful measures of association, access to a variety of statistical models, a statistical test that adjusts for multiple testing, and are easily automated in other software packages [13, 21, 32]. The choice of Poisson models for these scans allowed us to monitor for clusters in the rate of calls for the various sources and outcome measures considered. It also allowed for the inclusion of expected daily counts.

### Exposure-based surveillance

Retrospective temporal scans of pet food exposures reported to the APCC were successful in identifying the melamine contamination incident prior to the voluntary pet food recall. However, the ability of these scans to identify statistically significant clusters varied by call source.

Household and patient-level scans of the entire dataset and calls from the public performed equally well, regardless of covariate adjustment. These scans consistently identified statistically significant clusters of pet-food related calls beginning on March 13, 2007, three days prior to the voluntary recall by the pet food manufacturer. The duration of these clusters varied by level of analysis, with household-level clusters up to four months shorter in duration than patient-level clusters. However, the timing of the onset of these clusters concurs with reported increases in complaints to the FDA concerning potentially contaminated pet foods [7], suggesting these clusters could have been instrumental in the early detection of the pet food contamination incident.

Conversely, scans of calls from veterinary staff did not successfully identify any significant clusters prior to the date of the voluntary recall. Covariate month-adjustment did improve the ability of these scans to detect statistically significant clusters, although they were identified four days after the voluntary recall. The variation seen in scan performance across call sources likely reflects differences in reporting behaviours of veterinary staff and pet owners. Previous studies of APCC call data have noted variation in reporting rates [36, 37], common exposures [35], clinical signs, and severity measures [35] between call sources.

### Syndromic surveillance

Temporal scan performance varied substantially across the two chosen syndrome definitions with notably better performance seen for scans using MCTS calls compared to those using RDS calls. This finding supports our hypothesis that the MCTS definition would be more sensitive than the RDS definition at detecting the outbreak, as it included non-specific syndromic components, like vomiting and lethargy, associated with melamine-cyanuric acid toxicity [9]. The RDS definition was more specific, as it only included signs related to the urinary system, resulting in fewer clusters of shorter duration. However, choice of call source and covariate month-adjustment lead to very different results across both syndromes.

Unadjusted temporal scans of MCTS calls identified statistically significant clusters at both the household and patient level regardless of call source. These clusters were identified on March 13 and 20, for veterinary and public call data, respectively. These clusters are similar to those identified based on calls related to pet food suggesting these scans are identifying a similar signal. These were the only scans where a veterinary source detected a cluster earlier than a scan using all or only public calls.

The impact of covariate month-adjustment on the performance of MCTS scans vastly differed by call source. Covariate month-adjustment improved the performance of MCTS scans when the entire dataset or calls from the public were used. Prior to month adjustment, these scans were not successful in identifying the contamination incident prior to the voluntary recall. Whereas following adjustment, all MCTS clusters were identified in early January, up to 2.5 months earlier than the voluntary recall and 1.5 months prior to the first consumer complaint [8]. These clusters were also shorter in duration than the clusters identified using unadjusted scans. This adjustment effect, whereby the cluster size decreases as the amount of information in the model used for covariate adjustment increases, has been noted by other researchers as well [12, 21]. The onset of these clusters concurs with the timing of syndromic clusters previously identified by other researchers using alternative data sources and methodologies [17], as well as the timeline of the outbreak [8]. Consequently, the clusters identified could have been helpful in identifying there was an outbreak of serious renal failure which may have helped lead to the early detection of this pet food contamination incident.

Covariate adjustment was found to have the opposite effect on the performance of MCTS scans of calls from veterinary staff. Although unadjusted scans of MCTS syndromes reported by veterinary staff successfully identified syndromic clusters prior to the voluntary pet food recall, month-adjusted scans either did not identify any significant clusters or clusters which began in October, three months after the last case was reported to the FDA [8]. It is possible that this cluster identified a different outbreak as the timing coincides with the period when the FDA began receiving reports of Fanconi-like renal failure due to the consumption of pet jerky treats imported from China [38]. This is interesting since 60% of complaints reported gastrointestinal system involvement, and only 30% reported urinary system involvement and the MCTS includes gastrointestinal syndromic components.

These findings suggest that the MCTS definition which included non-specific syndromic components associated with melamine-cyanuric acid toxicosis such as diarrhea, anorexia, and lethargy, among others, may have increased the sensitivity of the scans. These findings contrast with those recently reported by other researchers, as they found the sensitivity of detecting the melamine incident decreased when non-specific syndromic components were analyzed compared to specific components like elevated creatine [17]. This contrast may be due to differences in the data sources and methodologies used in the two studies. Weng *et al*. (2020) analyzed the syndromic components separately, whereas in this present study, an any/or approach was utilized, which grouped all the syndromic components into one syndrome. A patient was classified as positive for the syndrome if there was a report of at least one of the syndromic components within the syndrome definition. There may also be differences in the way veterinary hospital EMR data are collected or analyzed compared to APCC call data which may bias or impact the performance of the syndromic scans. As the APCC generally receives more calls from the public than veterinary staff, non-specific syndromic components may be more useful for conducting surveillance using these data due to factors associated with reporting. Specifically, there are some syndromic components that are likely impossible for the public to report (e.g., laboratory results, clinical findings), and it is not guaranteed that veterinary staff will call back to report these measures. For example, an owner would not be able to report elevated creatine prior to their pet being seen by a veterinary service provider. This may result in the underreporting or underrepresentation of these more specific syndromic components in APCC call data compared to EMR data from veterinary clinics or hospitals. These factors should be considered prior to syndrome development and choosing an appropriate statistical methodology for conducting syndromic surveillance using APCC call data. Furthermore, it would be worthwhile to explore additional methodologies for analyzing these syndromic data, such as stratification [17], multivariate surveillance [19, 39], and parallel surveillance methods [39] in order to assess multiple syndromes or syndromic components at once.

When interpreting the results of this study it is important to note that APCC call data are highly sensitive to changes in popularity, use, and abundance of products in and around the home [34]. Furthermore, media coverage of foodborne outbreaks may increase the general public’s awareness of the APCC, which may lead to increased call volumes and possibly trigger surveillance alarms. The variation seen in the performance of scans based on surveillance outcome, statistical method and call source further demonstrates the importance of preliminary exploratory analyses and validation studies using simulated or documented outbreaks prior to integrating a standard cluster detection method into an existing surveillance system. Although the clusters identified in this study concur with the timeline of the outbreak as well as previous research, validation of identified outbreaks based on APCC call data is extremely difficult, as callers are not required to call back to report findings or outcomes and samples are not sent to the APCC for testing to confirm agent etiology. Future validation efforts could focus on other known outbreaks, such as the Fanconi-like renal failure syndrome outbreak in fall 2007 as a result of consumption of pet jerky treats [38]. Simulated outbreaks may also provide a useful method for validation, in order to assess or calculate possible false positive and false negative fractions (i.e., type I and II errors) of the potential surveillance system.

Integration of APCC call data with other sources of veterinary and human electronic medical record (EMR) data may be beneficial for both conducting surveillance and validating subsequent alarms. One source of EMR data that may be particularly beneficial for integration with APCC data are call data from regional PCs which are contained in the National Poison Data System, operated by America’s Poison Centers. Although regional PCs typically receive calls involving human exposures, approximately 1 in 20 calls involve potentially toxic exposures in pets [2]. Consequently, data from the National Poison Data System may help fill data gaps that result from lack of awareness of organizations like the APCC or where fees associated with animal-specific PCs impact access for certain individuals and/or communities. Additionally, veterinary EMR data from private veterinary hospitals and clinics may represent alternative sources of EMR data that may be beneficial for integration with APCC call data. Recently, researchers have demonstrated veterinary EMR data from private veterinary clinics and hospitals can be successfully used for identifying clusters of enteric [15, 16] and renal syndromes [16, 38] in companion animals. However, outbreak validation using data from these sources can still be difficult due to missing data or a lack of sufficient detail in veterinary records [15]. Furthermore, owner reported non-specific clinical signs, such as vomiting or lethargy, may reduce sensitivity of syndromic surveillance as many diseases and toxins can cause non-specific signs [38]. As surveillance alarms based on APCC call data, or other EMR data, likely reflect complex relationships between reporting and care seeking behaviours, patient-level factors, exposures and clinical signs, results from quantitative disease surveillance systems should be examined in concert with practitioners specialized in veterinary medicine and toxicology.

## Conclusions

This study demonstrates that APCC call data may be useful for quantitative disease surveillance in the US companion animal population. The success of the methods explored in this study in the early identification of the melamine contamination incident are promising. In addition, controlling for known monthly trends may represent the simplest method for improving temporal scan performance, by effectively controlling for some inherent noise in the dataset, when conducting syndromic surveillance using APCC call data. However, the adjustment approach did not always result in improved performance of the scans. Consequently, further validation is warranted.

This study provides additional evidence of the differences between calls to the APCC originating from the public and those originating from veterinary staff and illustrates the impact choice of call source can have on quantitative disease surveillance. These differences likely reflect a combination of several complex factors associated with reporting at the patient, owner, veterinary staff, and community level so the impact of call source should not be ignored when conducting surveillance using these data.

By combining model-based approaches with scan statistics, surveillance researchers can adjust for both categorical and continuous variables while locating clusters in space and/or time [12, 21, 40]. However, exploratory analyses to identify key factors that should be accounted for prior to applying quantitative surveillance methods are warranted since the results of scan statistics have been shown to vary substantially with covariate adjustment methods [12, 21]. We demonstrated adjusting for known monthly trends in APCC call data can lead to improved performance of temporal scan statistics. However, the impact of month adjustment on scan performance was found to vary by surveillance outcome and call source. These results caution against ignoring or haphazardly choosing covariates and adjustment methods when applying quantitative methods in surveillance applications. Although model adjusted methods have been shown to frequently perform better in surveillance applications, these methods are more complex to implement than standard aberration detection systems or running unadjusted scan statistics [12]. Consequently, the feasibility of the adoption, integration, and implementation of these methods into an existing surveillance system is dependent on the analytical and technical capacity and capability of the existing system and its operators.

## List of abbreviations

APCC: Animal Poison Control Center
AnTOX: Animal Toxin Database
ASPCA: American Society for the Prevention of Cruelty to Animals
EMR: electronic medical records
FDA: Food and Drug Administration
MCTS: melamine-cyanuric acid toxicosis syndrome
PC: poison center
RDS: renal dysfunction syndrome
US: United States of America
